# Pilot clinical evaluation of artificial intelligence–driven guiding catheter simulation for optimizing percutaneous coronary intervention

**DOI:** 10.1093/ehjdh/ztag021

**Published:** 2026-02-03

**Authors:** Masataka Yoshinaga, Hirooki Higami, Eiichi Watanabe, Takashi Muramatsu, Keisuke Murata, Toru Araki, Akane Miyazaki, Makoto Fujioka, Taishi Fukushima, Takehiro Ito, Tatsumasa Ueda, Yoshihiro Sobue, Wakaya Fujiwara, Kenya Nasu, Hitoshi Matsuo, Ken Kozuma, Hideo Izawa

**Affiliations:** Department of Cardiology, Fujita Health University Bantane Hospital, 3-6-10, Otobashi, Nakagawa-ku, Nagoya, Aichi 454-8509, Japan; Teikyo University Innovation Lab, Okinaga Research Institute, Tokyo, Japan; Department of Cardiology, Fujita Health University Bantane Hospital, 3-6-10, Otobashi, Nakagawa-ku, Nagoya, Aichi 454-8509, Japan; Department of Cardiology, Fujita Health University Hospital, Aichi, Japan; Department of Cardiology, Fujita Health University Bantane Hospital, 3-6-10, Otobashi, Nakagawa-ku, Nagoya, Aichi 454-8509, Japan; Department of Cardiology, Fujita Health University Bantane Hospital, 3-6-10, Otobashi, Nakagawa-ku, Nagoya, Aichi 454-8509, Japan; Department of Cardiology, Fujita Health University Bantane Hospital, 3-6-10, Otobashi, Nakagawa-ku, Nagoya, Aichi 454-8509, Japan; Department of Clinical Engineering, Fujita Health University Bantane Hospital, Aichi, Japan; Department of Cardiology, Nagoya University, Aichi, Japan; Department of Cardiology, Fujita Health University Bantane Hospital, 3-6-10, Otobashi, Nakagawa-ku, Nagoya, Aichi 454-8509, Japan; Department of Radiology, Fujita Health University Bantane Hospital, Aichi, Japan; Department of Cardiology, Fujita Health University Bantane Hospital, 3-6-10, Otobashi, Nakagawa-ku, Nagoya, Aichi 454-8509, Japan; Department of Cardiology, Fujita Health University Bantane Hospital, 3-6-10, Otobashi, Nakagawa-ku, Nagoya, Aichi 454-8509, Japan; Department of Cardiology, Mie Heart Center, Mie, Japan; Department of Cardiovascular Medicine, Gifu Heart Center, Gifu, Japan; Division of Cardiology, Teikyo University Hospital, Tokyo, Japan; Department of Cardiology, Fujita Health University Hospital, Aichi, Japan

**Keywords:** Artificial intelligence, Innovation, Percutaneous coronary intervention, Guiding catheter simulation, Coronary computed tomography angiography, Chronic coronary syndrome

## Abstract

**Aims:**

In percutaneous coronary intervention (PCI), a suboptimal choice of guiding catheter may compromise coaxial alignment and backup support, prolonging procedures and increasing radiation and contrast exposure. We assessed whether a computed tomography (CT)–driven, artificial intelligence (AI)–guided preprocedural simulation could improve procedural efficiency and safety.

**Methods and results:**

In a single-centre prospective registry with historical controls, 55 consecutive elective procedures performed with CT-based AI-assisted guiding-catheter selection were compared with 55 procedures performed without assistance. The primary endpoint was total procedure time from arterial access to completion. Secondary endpoints included time to coronary engagement, radiation dose, contrast volume, and guiding-catheter-related events. Computed tomography–-based AI assistance was associated with shorter procedures (mean 68.5 vs. 91.8 min), shorter engagement time, lower radiation dose, and lower contrast use. Guiding-catheter exchanges were fewer, and catheter-related events were lower (3.6 vs. 16.4%; risk ratio 0.22; 95% confidence interval 0.05–0.98). Procedural success was 100% in both groups with no in-hospital major adverse cardiac or cerebrovascular events.

**Conclusion:**

A CT-driven, CT-based AI-guided simulation for guiding-catheter selection was associated with greater procedural efficiency and a favourable profile in elective PCI. This approach, which standardizes catheter choice and is associated with fewer empirical catheter exchanges, warrants confirmation in multicentre randomized studies and may help optimize resource utilization in routine PCI.

## Graphical Abstract

**Graphical Abstract:**
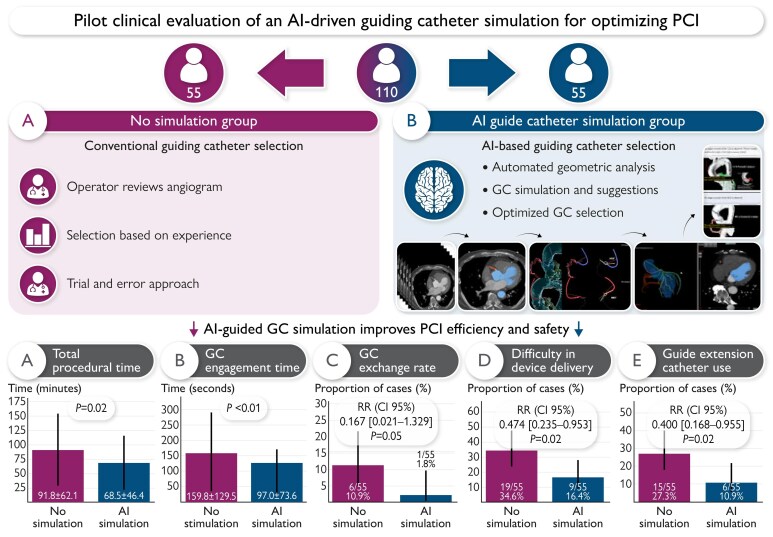
AI-guided vs. conventional guiding catheter selection in PCI. Visual summary comparing conventional planning vs. an AI-guided, CT-driven preprocedural simulation for guiding-catheter selection. In conventional practice, catheter choice is based on operator judgement and may require multiple exchanges; the AI system integrates coronary CT to simulate engagement and recommend an initial guiding catheter. In this prospective registry, AI assistance was associated with shorter procedures and engagement times, lower radiation dose and contrast volume, fewer catheter exchanges, and fewer catheter-related events, while maintaining 100% procedural success. Numerical estimates are provided in the main text and tables. AI, artificial intelligence; CT, computed tomography; GC, guiding catheter.

## Introduction

Percutaneous coronary intervention (PCI) is a cornerstone therapy for ischaemic heart disease, and procedural success largely depends on the appropriate selection and manipulation of the guiding catheter (GC). In particular, for complex lesions such as chronic total occlusions (CTO), GC selection must account for the anatomical characteristics of the aorta and coronary arteries to ensure coaxial alignment and sufficient backup support. However, it is often difficult to fully appreciate the three-dimensional relationship between devices and anatomy, and GC selection in current practice largely relies on operator experience and estimation. In recent years, efforts have been made to incorporate three-dimensional information from pre-procedural coronary computed tomography (CT) into PCI planning,^1,2^ and current guidelines recommending coronary CT as the first-line diagnostic modality for stable coronary artery disease^3^ further encourage this approach. Nevertheless, when the guiding-catheter shape and size do not adequately match the patient’s anatomy, selective engagement and backup support may be suboptimal. In such cases, operators often need to adjust catheter depth and manipulation, use guide-extension support, or change to alternative catheter shapes, and in more complex anatomies, multiple catheter exchanges may ultimately be required. Such mismatches can prolong the procedure, increase radiation exposure and contrast volume, and ultimately raise the risk of procedural failure. Recently, Higami and colleagues^4,5^ developed an innovative tool—the virtual reality (VR) guiding catheter simulation system—aimed at standardizing GC selection for PCI in patients with chronic coronary syndrome (CCS) and enabling safer, more predictable completion of complex procedures. Furthermore, leveraging artificial intelligence (AI) which is increasingly expected to enhance efficiency, diagnostic accuracy, reduce physician workload, and mitigate regional disparities in healthcare delivery,^6^ the GUIDEYE system was developed as a novel AI-based GC selection application that automatically analyses morphological information of the aorta and coronary arteries from CT images to propose the optimal GC (*Graphical Abstract*). In previous work, Higami and colleagues reported a virtual reality (VR)–based guiding catheter simulation system primarily designed for operator training and manual virtual manipulation of catheters. In contrast, the present GUIDEYE platform implements a fully automated, CT-based artificial intelligence (AI) pipeline that analyses pre-procedural coronary CT and directly outputs a recommended guiding-catheter type and size for use in routine PCI. Given these advancements, it became necessary to validate in clinical practice whether pre-procedural AI-based GC selection, performed by GUIDEYE through automated CT analysis and engagement simulation, aligns with the GCs ultimately used during PCI. It is anticipated that enabling optimal GC selection pre-procedurally may enhance procedural efficiency by shortening procedure times and decreasing unnecessary device use, contrast volume, and radiation exposure. Demonstrating the safety and efficacy of this approach in real-world settings may support AI-based GC selection GUIDEYE as a useful adjunct for ensuring safe and efficient planned PCI in CCS. In this study, we compared procedural outcomes between PCI cases in which GCs were selected conventionally based on operator experience and pre-procedural coronary CT and angiographic information without AI assistance and those in which GCs were selected using pre-procedural AI-assisted GUIDEYE analysis.

## Methods

### Study design and population

This was a prospective registry with non-randomized controls, a single-centre interventional study conducted at Fujita Health University Bantane Hospital (Aichi), Japan. Eligible patients were those undergoing elective PCI between 1 October 2024 and 8 August 2025 who had undergone pre-procedural coronary CT, excluding those meeting predefined exclusion criteria. At our institution, pre-procedural GC simulation using GUIDEYE was initiated on 1 October 2024 for elective PCI. Between 1 October 2024 and 17 January 2025, GUIDEYE simulation was performed in 36 pilot cases. Following institutional review board (IRB) approval on 17 January 2025, consecutive elective PCI cases in which pre-procedural AI simulation was used for GC selection and written informed consent was obtained were prospectively enrolled as the AI-simulation group (*n* = 55) between 17 January and 6 June 2025. The control group (No-simulation group) comprised consecutive elective PCI cases performed between 1 October 2024 and 8 August 2025 at the study centre in which pre-procedural coronary CT data were available, but GC selection was based solely on the operator’s experience using CT and angiographic information, without running the GUIDEYE simulation. Importantly, there was no random allocation between the AI-simulation and No-simulation groups. Each procedure could only belong to one group, and there was no within-patient or within-procedure randomization. Among 234 consecutive elective PCI cases performed during this period, we excluded pilot GUIDEYE cases (*n* = 36), PCI without informed consent (*n* = 7), procedures without pre-procedural coronary CT (*n* = 42), cases performed using other angiography systems (*n* = 11), CTO cases (*n* = 28), and the AI-simulation group (*n* = 55), resulting in 55 cases included in the No-simulation group. This study was approved by the IRB of Fujita Health University (approval number HM24-331) and conducted in accordance with the Declaration of Helsinki.

### GUIDEYE simulation

**Figure 1:**
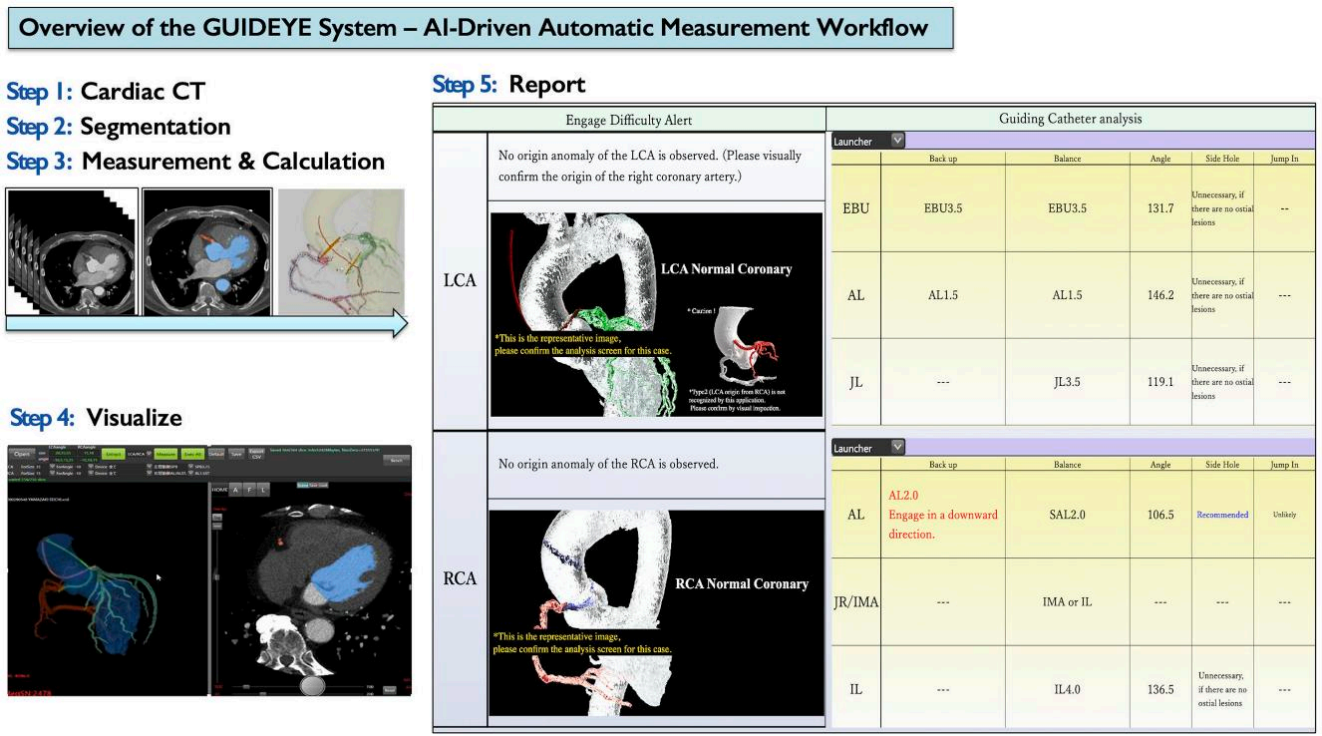
Workflow of artificial intelligence–based, computed tomography–driven guiding-catheter simulation (GUIDEYE). Baseline coronary computed tomography angiography is acquired before elective percutaneous coronary intervention. The aortic root, coronary ostia, and trajectories of the coronary arteries are automatically segmented by the artificial intelligence–driven algorithm. A virtual three-dimensional aortic root model is reconstructed, and candidate guiding catheter shapes are overlaid along the coronary ostium. The system simulates catheter engagement from the arterial access route, estimating coaxial alignment, back-up support, and risk of deep intubation and report generation within 5–7 min. The operator can interactively review alternative catheter options on a dedicated workstation before the procedure. Report shows an enlarged view of the GUIDEYE output screen with the AI-recommended guiding catheter type and size, back-up score, predicted tip position, and warnings for deep engagement risk. 3D, three-dimensional; CT, computed tomography; GC, guiding catheter; PCI, percutaneous coronary intervention.

The newly developed DICOM workstation GUIDEYE (Anreal Twin Ltd, Gifu, Japan; founded by Hirooki Higami and Kazuhiko Matsumoto) implements a PCI guiding-catheter simulation that is fundamentally based on machine-learning techniques for anatomical tissue region extraction. Coronary CT angiography datasets are imported into the CT-based AI workstation, which automatically analyses the axial thin-slice DICOM data. From each cardiac CT dataset, the system automatically identifies the right and left coronary arteries, the Valsalva sinuses, the three aortic valve cusps, the left ventricle, the aortic valve commissures, the sinotubular junction (STJ), the aortic centreline, and the coronary centrelines. It segments the aortic root and coronary ostia, extracts a three-dimensional centreline of the target vessel, and identifies the anticipated guiding-catheter landing zone. A deep-learning model trained on annotated cases then predicts the guiding-catheter type and size expected to provide coaxial alignment and sufficient backup support, based on aortic root dimensions, coronary take-off angle, and STJ height. The entire process—from CT data analysis to report generation—requires no manual correction and is completed within ∼5–7 min (*Figure 1*).

**Figure 2:**
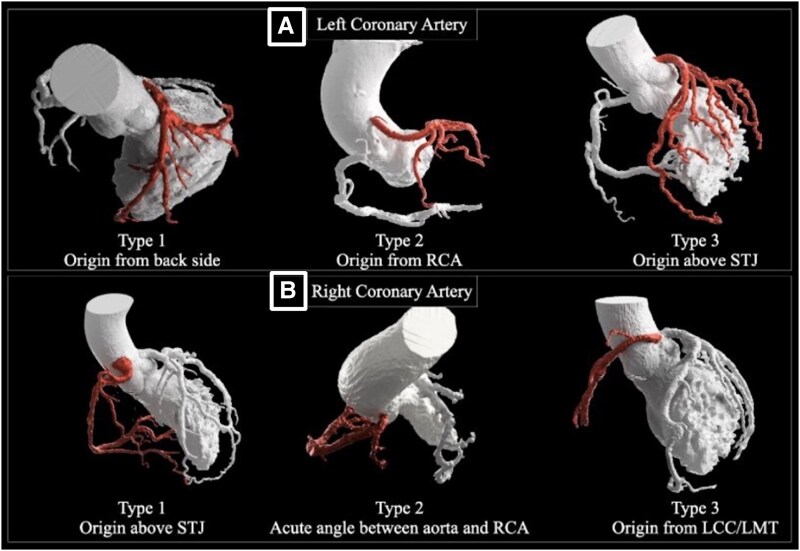
Three-dimensional rendering images of anatomical classification of the coronary arteries. Representative three-dimensional renderings of aortic root and coronary take-off variants (e.g. ostial height, sinus size, commissural orientation) that alter coaxial alignment and backup support. The simulation incorporates these phenotypes to prioritize guiding catheter shape and size most likely to achieve stable engagement. Representative three-dimensional computed tomography renderings showing six anatomical subtypes for left coronary artery and right coronary artery origins. 3D, three-dimensional; AO, aortic root; CT, computed tomography; GC, guiding catheter; LCA/RCA, left/right coronary artery; LCC, left coronary cusp; LMT, left main trunk; STJ, sinotubular junction.

Additionally, the system calculates the angle from the right and left commissures to each coronary ostium, thereby recognizing origins such as dorsal left coronary artery (LCA), anterior right coronary artery (RCA), and left-cusp origins. On this basis, it classifies six patterns associated with challenging engagement (*Figure 2*). Various guiding-catheter shapes and sizes are preregistered in a three-dimensional model database. Currently, the simulation is compatible with two guiding-catheter platforms: Hyperion (ASAHI Intecc, Aichi, Japan) and Launcher (Medtronic, Minneapolis, MN, USA). The application integrates patient-specific anatomical data with this database to automatically determine the recommended catheter shape and size. For the RCA, operators can select between Amplatz Left-type catheters (AL, AL Short Tip, or SAL) and Judkins Right-type catheters (JR 3.5, JR 4.0, or IM), with optimal size and shape suggestions provided for each. For the LCA, operators can choose from Judkins Left-type catheters (JL 3.5, JL 4.0), backup-type catheters (SPB 3.0, 3.5, 3.75, 4.0 or EBU 3.5, 4.0), or AL-type shapes, with corresponding size recommendations. The operator can review the three-dimensional rendering and virtual catheter overlay and may either accept or override the suggested catheter. In the AI-simulation group, operators reviewed the simulation report preprocedurally and made the final guiding-catheter selection considering angiographic findings and patient background; in those cases, the AI-recommended catheter was used as the initial guiding catheter.

### Percutaneous coronary intervention procedure

All procedures were performed using the same angiography system (Alphenix Evolve Edition, Canon Medical Systems, Tokyo, Japan). Six- to eight French GCs were used via radial, femoral, or brachial access. Patients received aspirin plus clopidogrel or prasugrel, and unfractionated heparin to maintain an ACT >300 s.^7^ After wiring the target lesion, PCI was performed according to standard techniques.^8,9^ Intravascular ultrasound (IVUS) guidance was mandatory.^9,10^ The use of guide extension catheters, atherectomy devices [rotational atherectomy (RA), orbital atherectomy, or intravascular lithotripsy], scoring/cutting balloons, and mechanical circulatory support (MCS) was at the operator's discretion.^(11,12)^

### Study endpoints

The primary endpoint was total procedure time (minutes), defined as the interval from introduction of the first guiding catheter into the aortic root to completion of PCI,^13^ i.e. acquisition of the final coronary angiographic run after stent/balloon delivery and removal of all intracoronary devices and the guiding catheter. Time required for vascular closure and removal of the arterial sheath was not included. Secondary endpoints were procedural success rate [defined as successful stent or device delivery with residual stenosis <30% by visual estimation and no in-hospital major adverse cardiac or cerebrovascular events (MACCE)], number of GCs used per case, GC engagement time (from sheath insertion to successful coronary engagement), contrast volume used,^14^ radiation exposure (reference peak skin dose and air kerma),^15^ fluoroscopy time, incidence of device delivery difficulty, guide-extension catheter usage rate,^16^ and GC-related events. Safety outcomes were GC-related events (including wedge position, pressure damping, and ostial dissection) and other periprocedural complications such as cardiac tamponade, coronary perforation, stroke, emergency surgery, and in-hospital major adverse cardiac and cerebrovascular events. Device-delivery difficulty was defined as the need for additional support techniques (e.g. guide-extension catheter, anchor balloon, deep engagement, or buddy wire) to deliver balloons or stents to the target lesion after successful GC engagement, or failure to deliver a planned device. Guiding catheter-related events were defined as adverse events attributable to the guiding catheter, including ostial coronary dissection, new or worsened aortic regurgitation (AR) judged to be catheter-induced, and wedge or deeply intubated catheter position associated with haemodynamic damping. Transient pressure damping or wedge position that resolved promptly after slight catheter withdrawal and did not lead to ischaemic electrocardiogram (ECG) changes or haemodynamic instability was still recorded as a GC-related haemodynamic event, but interpreted as a minor component of the composite endpoint. All GC-related events and device-delivery difficulty were prospectively recorded by the operator and verified offline by an independent interventional cardiologist blinded to group allocation using angiography, haemodynamic waveforms, and ECG; discrepancies were resolved by consensus. Radiation dose parameters, including reference air kerma (mGy) and peak skin dose (mGy), were automatically recorded by the Alphenix Evolve Edition X-ray system (Canon Medical Systems) at the interventional reference point and calculated by the system's integrated dosimetry software and exported from the dose report.

### Statistical analysis

Continuous variables are presented as mean ± standard deviation and categorical variables as counts and percentages. Groups were compared using Student’s *t*-test for continuous and *χ*^2^ or Fisher’s exact test for categorical variables. Statistical significance was set at *P* < 0.05. To reduce selection and temporal bias, prespecified multivariable adjustment and inverse probability of treatment weighting (IPTW) with stabilized weights were applied; covariate balance was assessed by standardized mean differences (<0.10). Analyses were performed using JMP Pro 17 (SAS Institute Inc., Cary, NC, USA). Secondary endpoints were exploratory and not adjusted for multiplicity; 95% confidence intervals (CIs) are provided to aid interpretation. We used IPTW with stabilized weights based on a logistic regression including age, prior heart failure, SYNTAX score, lesion length, access site, operator experience, and calendar month; covariate balance was assessed by standardized mean differences (<0.10). Calendar month of the procedure was included as a covariate to adjust for potential secular trends in case mix, operator experience, and workflow changes over the study period. Missing data were handled using multiple imputation.

## Results

### Study population

**Figure 3:**
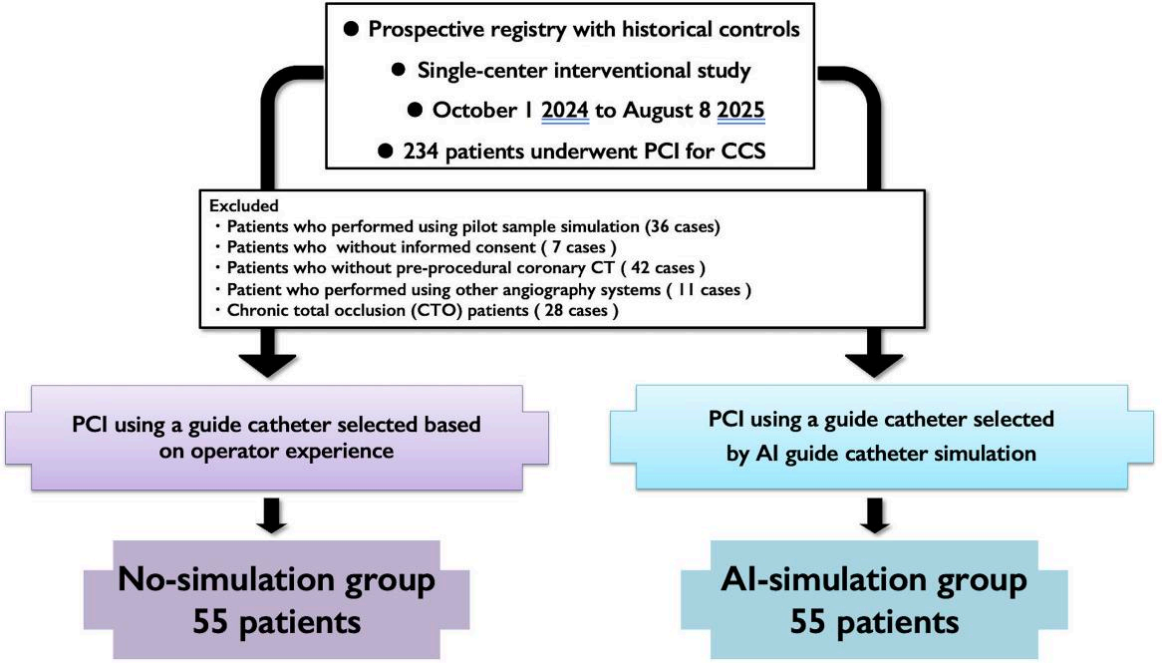
Study flowchart of patient selection and group allocation. Patient enrolment and allocation: 234 screened, 110 analysed (No-simulation: 55, AI-simulation: 55). Screening, predefined exclusions, and allocation to artificial intelligence–assisted vs. non-assisted groups are depicted. Consecutive elective cases were analysed after exclusions; all procedures used contemporary intravascular imaging guidance. Final analytic cohorts are shown at the bottom of the diagram. CCS, chronic coronary syndrome.

Of 234 patients with CCS who underwent elective PCI during the study period, 110 consecutive cases (47.0%) met the inclusion criteria and were analysed: 55 in the AI-simulation group (pre-procedural simulation with AI GUIDEYE) and 55 in the No-simulation group. The study flow is shown in *Figure 3*.

### Baseline characteristics

Baseline patient and lesion characteristics are summarized in *Tables 1* and *2*. Age, comorbidities, and angiographic lesion complexity were well balanced between groups (all *P* ≥ 0.10). SYNTAX score (16.6 ± 8.1 vs. 15.8 ± 8.6; *P* = 0.65) and the proportion of American College of Cardiology/American Heart Association type C lesions [76.3% (*n* = 42/55) vs. 67.2% (*n* = 37/55); *P* = 0.32] were similar. The rate of *ad hoc* PCI was numerically higher in the AI-simulation group than in the non-simulation group [29.1% (*n* = 16/55) vs. 20.0% (*n* = 11/55); *P* = 0.26]. Importantly, operator PCI experience was comparable between groups [<100 cases: 3.6% (*n* = 2/55) vs. 5.4% (*n* = 3/55); 100–300 cases: 67.3% (*n* = 37/55) vs. 67.3% (*n* = 37/55); >300 cases: 29.1% (*n* = 16/55) vs. 20.0% (*n* = 11/55); *P* = 0.89], indicating that procedural outcomes were not confounded by operator experience. All procedures were performed under IVUS guidance.

**Table 1:** Patient and lesion characteristics

Variables	No simulation group (*N* = 55)	AI simulation group (*N* = 55)	*P*-value
Age, year	72.1 ± 9.7	75.3 ± 9.1	0.07
Male gender, *n* (%)	41 (74.5)	39 (70.9)	0.66
Body mass index, kg/m^2^	23.8 ± 3.8	23.9 ± 4.3	0.88
Hypertension, *n* (%)	49 (89.1)	46 (83.6)	0.40
Diabetes mellitus, *n* (%)	24 (43.6)	28 (50.9)	0.44
Dyslipidaemia, *n* (%)	51 (92.7)	49 (89.0)	0.50
Current smoker, *n* (%)	14 (25.4)	14 (25.4)	1.00
LEAD, *n* (%)	14 (25.4)	19 (34.5)	0.29
Haemodialysis, *n* (%)	8 (14.5)	7 (12.7)	0.78
History of stroke, *n* (%)	18 (32.7)	16 (29.0)	0.17
Family history of CAD, *n* (%)	9 (16.3)	15 (27.2)	0.10
Prior percutaneous coronary intervention, *n* (%)	27 (49.1)	24 (43.6)	0.56
Prior bypass graft surgery, *n* (%)	1 (1.8)	1 (1.8)	1.00
Prior myocardial infarction, *n* (%)	11 (20.0)	15 (27.2)	0.36
Prior heart failure, *n* (%)	9 (16.3)	18 (32.7)	0.04
History of TEVAR/EVAR	0 (0.0)	0 (0.0)	N/A
History of aortic replacement	0 (0.0)	0 (0.0)	N/A
Atrial fibrillation, *n* (%)	6 (10.9)	4 (7.2)	0.50
Systolic blood pressure, mmHg	159.7 ± 29.1	161.9 ± 32.2	0.71
Diastolic blood pressure, mmHg	78.6 ± 16.6	78.7 ± 16.9	0.97
LVEF, %	54.2 ± 10.1	52.8 ± 11.7	0.51
Aortic regurgitation, *n* (%)	9 (16.3)	15 (27.2)	0.16
Aortic stenosis, *n* (%)	2 (3.6)	5 (9.0)	0.24
Haemoglobin, g/dL	12.8 ± 1.6	12.6 ± 1.8	0.54
Platelet, ×10^4^/μL	21.2 ± 6.6	20.4 ± 5.5	0.48
eGFR, mL/min/1.73 m^2^	56.7 ± 30.3	51.4 ± 27.0	0.33
eGFR (<60 mL/min/1.73 m^2^)	31 (56.3%)	34 (61.8%)	0.56
Haemoglobin A1c, %	6.6 ± 1.2	6.6 ± 1.4	0.82
Triglyceride, mg/dL	136.7 ± 66.6	116.7 ± 67.4	0.12
HDL-C, mg/dL	48.8 ± 15.5	51.4 ± 12.8	0.34
LDL-C, mg/dL	73.6 ± 33.6	81.3 ± 37.7	0.25
Clinical presentation, *n* (%)			0.58
Asymptomatic	9 (16.3)	7 (12.7)	
Stable angina	46 (83.6)	48 (87.2)	
Target vessel, *n* (%)			0.18
LMT	1 (1.8)	0 (0.0)	
RCA	17 (30.9)	17 (30.9)	
LAD	24 (43.6)	27 (49.0)	
LCX	11 (20.0)	6 (10.9)	
LMT–LAD	1 (1.8)	1 (1.8)	
LMT–LAD–LCX	0 (0.0)	1 (1.8)	
LAD–LCX	1 (1.8)	3 (5.4)	
Multivessel disease, *n* (%)	40 (72.7)	42 (76.3)	0.66
Medications on admission, *n* (%)			
Aspirin	55 (100.0)	55 (100.0)	1.00
P2Y12 inhibitors	55 (100.0)	55 (100.0)	1.00
Oral anticoagulants	7 (12.7)	5 (9.0)	0.54
ACE inhibitors, ARB, or ARNI	34 (61.8)	36 (65.4)	0.69
β-Blockers	35 (63.6)	35 (63.6)	1.00
Calcium channel blockers	24 (43.6)	18 (32.7)	0.23
Statins	54 (98.1)	50 (90.9)	0.09

Baseline demographics and lesion features.

ACE, angiotensin-converting enzyme; ARB, angiotensin receptor blockers; ARNI, angiotensin receptor neprilysin inhibitor; CAD, coronary artery diseases; eGFR, estimated glomerular filtration rate; EVAR, endovascular aneurysm repair; HDL-C, HDL cholesterol; LAD, left anterior descending coronary artery; LCX, left circumflex coronary artery; LEAD, lower extremity artery disease; LDL-C, LDL cholesterol; LMT, left main trunk; LVEF, left ventricular ejection fraction; MI, myocardial infarction; RCA, right coronary artery; TEVAR, thoracic endovascular aortic repair.

**Table 2:** Procedural characteristics and lesion complexity

Variables	No simulation group (*N* = 55)	AI simulation group (*N* = 55)	*P*-value
PCI operator status, *n* (%)			0.89
<100 cases	3 (5.4)	2 (3.6)	
100–300 cases	37 (67.3)	37 (67.3)	
300< cases	15 (27.3)	16 (29.1)	
*Ad hoc* PCI, *n* (%)	11 (20.0)	16 (29.1)	0.26
Vascular access, *n* (%)			0.91
Right radial	44 (80.0)	44 (80.0)	
Left radial	2 (3.6)	3 (5.5)	
Right brachial	2 (3.6)	1 (1.8)	
Left brachial	0 (0)	0 (0)	
Femoral	7 (12.7)	7 (12.7)	
Size of guiding catheter, *n* (%)			0.42
6 Fr	13 (23.6)	16 (29.1)	
7 Fr	42 (76.4)	39 (70.9)	
8 Fr	1 (1.8)	0 (0)	
Intracoronary imaging modalities, *n* (%)			
IVUS	55 (100.0)	55 (100.0)	1.00
Lesion severity scores			
SYNTAX SCORE	15.8 ± 8.6	16.6 ± 8.1	0.65
SYNTAX II score	38.5 ± 13.5	42.4 ± 15.2	0.16
SYNTAX II 4-year mortality (%)	20.5 ± 21.6	26.2 ± 25.4	0.21
Lesion length (mm)	35.5 ± 17.9	39.6 ± 21.3	0.26
Anatomical characteristics, *n* (%)			
Aortic ostium involvement	1 (1.8)	2 (3.6)	0.55
LCA ostium involvement	2 (3.6)	2 (3.6)	1.00
Bifurcation lesions	26 (47.3)	30 (54.6)	0.44
Diffuse disease	24 (43.6)	26 (47.2)	0.70
Heavy calcification	22 (40.0)	20 (36.4)	0.69
Vessel tortuosity	23 (41.8)	18 (32.7)	0.32
ACC/AHA lesion type, *n* (%)			0.32
A	0 (0.0%)	0 (0)	
B1	1 (1.8%)	0 (0)	
B2	17 (30.9)	13 (23.6)	
C	37 (67.2)	42 (76.3)	

Lesion complexity and procedural details.

ACC, American College of Cardiology; AHA, American Heart Association; IVUS, intravascular ultrasound; OCT, optical coherence tomography; PCI, percutaneous coronary intervention.

### Procedural outcomes and efficiency

**Figure 4:**
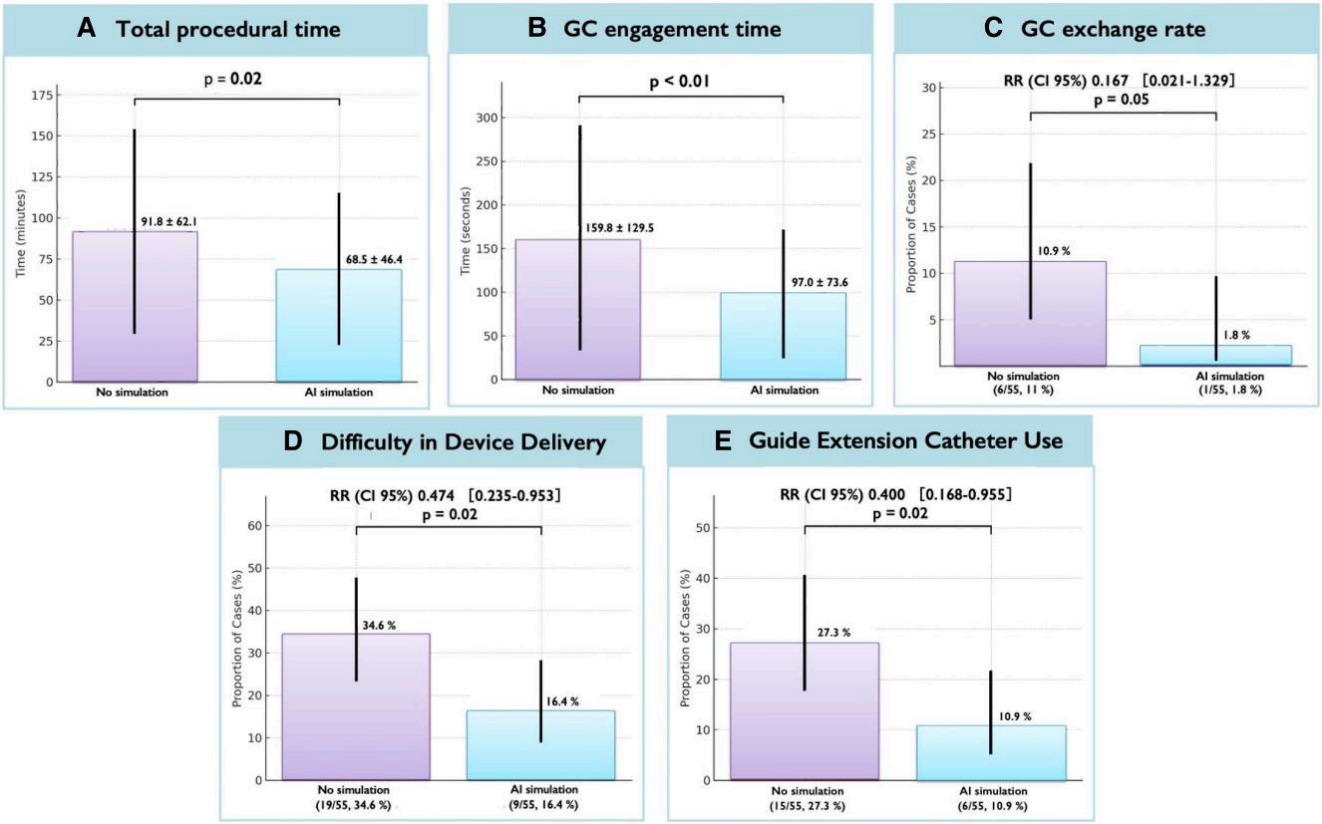
Procedural efficiency and device-support indicators. Compared with standard care, artificial intelligence assistance was associated with shorter total procedure and engagement times, fewer first-attempt mismatches requiring guiding catheter exchange, and lower rates of delivery difficulty and guide-extension use. Exact effect sizes and confidence intervals are reported in *Table 3*. GC, guiding catheter; Error bars denote 95% confidence intervals where shown; †inverse probability of treatment weighting-adjusted comparison.

Procedural outcomes are shown in *Table 3*. The primary endpoint of total procedure time was significantly shorter in the AI-simulation group (68.5 ± 46.4 vs. 91.8 ± 62.1 min; −23.3 min; 95% CI −43.8 to −2.8; *P* = 0.02) (*Figure 4A*). Procedural success was 100% in both groups. However, AI-simulation was associated with shorter GC engagement time and markers of greater procedural efficiency. Guiding catheter engagement time (from sheath insertion to successful coronary engagement) was significantly shorter in the AI-simulation group (97.0 ± 73.6 vs. 159.8 ± 129.5 s; mean difference −39.0 s; 95% CI −66.4 to −11.6; *P* < 0.01) (*Figure 4B*). The number of GCs used per case was slightly lower in the AI-simulation group (1.0 ± 0.1 vs. 1.1 ± 0.3; *P* = 0.05). Guiding catheter exchange due to failed initial engagement occurred in six cases in the No-simulation group but only one case in the AI-simulation group [1.8 vs. 10.9%; relative difference of 83% (relative risk (RR) 0.17); *P* = 0.05] (*Figure 4C*). The frequency of operator-reported device delivery difficulty was significantly lower with AI-simulation: 16.4% in the AI-simulation group vs. 34.6% in the No-simulation group (RR 0.474, 95% CI 0.235–0.953, *P* = 0.02) (*Figure 4D*). Guide-extension catheter use was also lower in the AI-simulation group than in the non-simulation group (10.9 vs. 27.3%; RR 0.40, 95% CI 0.17–0.96, *P* = 0.02) (*Figure 4E*). Side-hole guiding catheters were used in 44/55 (80.0%) procedures in the AI-simulation group and 33/55 (60.0%) procedures in the non-simulation group (*P* = 0.02).

**Table 3:** Procedural efficiency and device performance

Variables	No simulation group (*N* = 55)	AI simulation group (*N* = 55)	Mean difference (95% CI)	RR (95% CI)	*P*–value
PCI procedure success, *n* (%)	55 (100.0)	55 (100.0)			1.00
Number of guiding catheters used	1.1 ± 0.3	1.0 ± 0.1			0.05
Number of guiding catheter exchanges, *n* (%)				0.167 (0.021, 1.339)	0.05
0	49 (89.0)	54 (98.2)			
1	6 (10.9)	1 (1.8)			
2	0 (0)	0 (0)			
Guiding catheter engagement time (s)	159.8 ± 129.5	97.0 ± 73.6	(−102.2, −23.4)		<0.01
Side-hole guiding catheter use, *n* (%)	33 (60.0)	44 (80.0)			0.02
Number of guide wires, *n* (%)	1.8 ± 1.1	1.8 ± 0.8			0.77
Pre-dilation, *n* (%)	55 (100.0)	55 (100.0)			1.00
Number of pre-dilation balloons, *n* (%)	2.7 ± 1.1	2.5 ± 1.1			0.56
Pre-dilation balloon, *n* (%)					0.09
Conventional balloon	5 (9.1)	1 (1.8)			
Modified balloon	50 (90.9)	54 (98.2)			
PCI procedure final device, *n* (%)					
DES only therapy	32 (58.2)	35 (63.6)			0.55
DCB only therapy	7 (12.7)	4 (7.3)			0.34
DES and DCB hybrid therapy	16 (29.1)	16 (29.1)			1.00
Number of stents	1.5 ± 1.1	1.4 ± 0.8			0.84
Stent diameter, mm	(*N* = 48)	(*N* = 51)			
	3.2 ± 0.5	3.1 ± 0.5			0.55
Stent length, mm	(*N* = 48)	(*N* = 51)			
	41.5 ± 24.1	38.1 ± 22.8			0.47
Number of DCB, *n* (%)	0.5 ± 0.8	0.4 ± 0.7			0.61
DCB diameter, mm	(*N* = 23)	(*N* = 20)			
	2.5 ± 0.4	2.4 ± 0.5			0.59
DCB length, mm	(*N* = 23)	(*N* = 20)			0.55
	22.6 ± 9.6	24 ± 16.9			0.73
Rotational atherectomy, *n* (%)	7 (12.7)	4 (7.2)			0.34
Orbital atherectomy, *n* (%)	0 (0.0)	0 (0.0)			N/A
Directional coronary atherectomy, *n* (%)	0 (0.0)	0 (0.0)			N/A
Difficulty in device delivery, *n* (%)	19 (34.6)	9 (16.4)		0.474 (0.235, 0.953)	0.02
Guide extension catheter, *n* (%)	15 (27.3)	6 (10.9)		0.400(0.168, 0.955)	0.02
Anchor balloon technique, *n* (%)	2 (3.6)	0 (0.0)			0.15
Procedure time, min	91.8 ± 62.1	68.5 ± 46.4	(−43.8, −2.8)		0.02
Total volume of heparin, U	7.7 ± 2.4	6.7 ± 1.5	(−1.75, −0.25)		<0.01
Radiation time, min	32.2 ± 23.6	25.5 ± 19.8	(−14.8, 1.4)		0.10
Total radiation dose, mGy	1043.6 ± 708.6	683.7 ± 506.0	(−590.0, −129.8)		<0.01
Peak skin dose, mGy	776.9 ± 625.2	481.6 ± 367.8	(−487.0, −103.6)		<0.01
Contrast medium volume, mL	154.7 ± 76.1	124.4 ± 59.1	(−55.8, −4.8)		0.02

Engagement/procedure times, radiation/contrast, device difficulty, guide extension.

DCB, drug-coated balloon; DES, drug-eluting stents; PCI, percutaneous coronary intervention; RR, relative risk.

### Radiation exposure and contrast use

**Figure 5:**
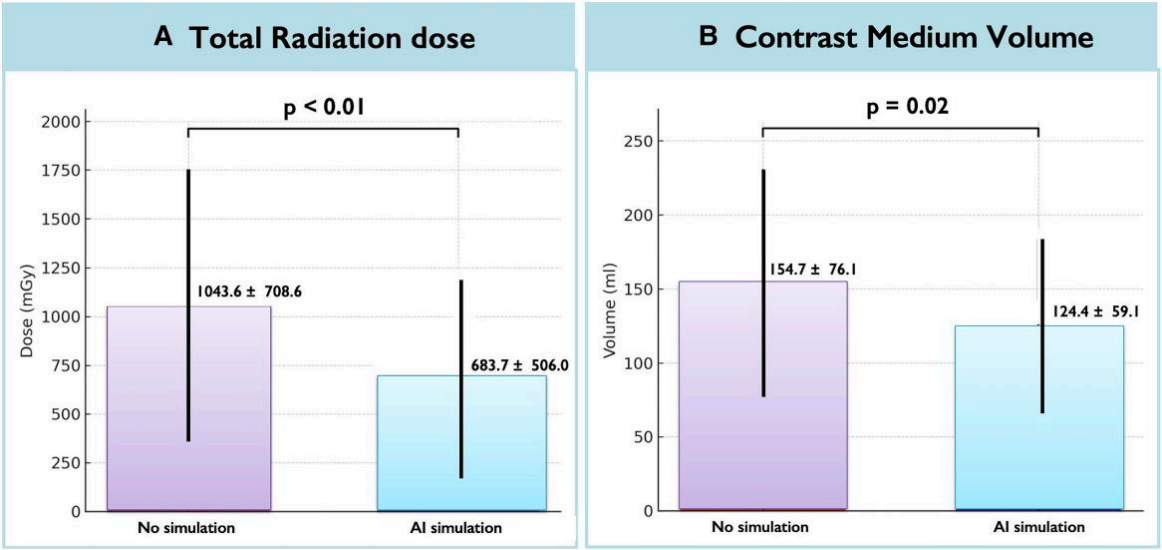
Radiation exposure and contrast utilization. With artificial intelligence assistance, radiation exposure metrics and contrast volume were lower than with conventional planning. Detailed means, differences, or ratios with 95% confidence intervals, and *P*-values are provided in *Table 3* and the Supplementary material online. Error bars denote 95% confidence intervals where shown.

Total radiation dose was lower in the AI-simulation group (air kerma: 683.7 ± 506.0 vs. 1043.6 ± 708.6 mGy; −360 mGy; 95% CI −590 to −129.8; *P* < 0.01) (*Figure 5A*) and peak skin dose was also lower in the AI-simulation group (481.6 ± 367.8 vs. 776.9 ± 625.2 mGy; −295 mGy; 95% CI −487 to −103.6; *P* < 0.01). Contrast volume was also lower in the AI-simulation group (124.4 ± 59.1 vs. 154.7 ± 76.1 mL; *P* = 0.02) (*Figure 5B*). There was a trend towards shorter fluoroscopy time (25.5 ± 19.8 vs. 32.2 ± 23.6 min; *P* = 0.10).

### Device strategy

Stent and drug-coated balloon (DCB) strategies were comparable between groups (drug-eluting stent (DES) only: 63.6 vs. 58.2%; DCB only: 7.3 vs. 12.7%; DES + DCB: 29.1 vs. 29.1%; all *P* > 0.30). The number, diameter, and length of DES/DCB used were similar. Atherectomy rates were also similar, though RA use was numerically higher in the No-simulation group [7.2% (*n* = 4/55) vs. 12.7% (*n* = 7/55); *P* = 0.34]. There were no differences between groups in the number of additional guidewires or balloons used.

### Safety and clinical outcomes

**Figure 6:**
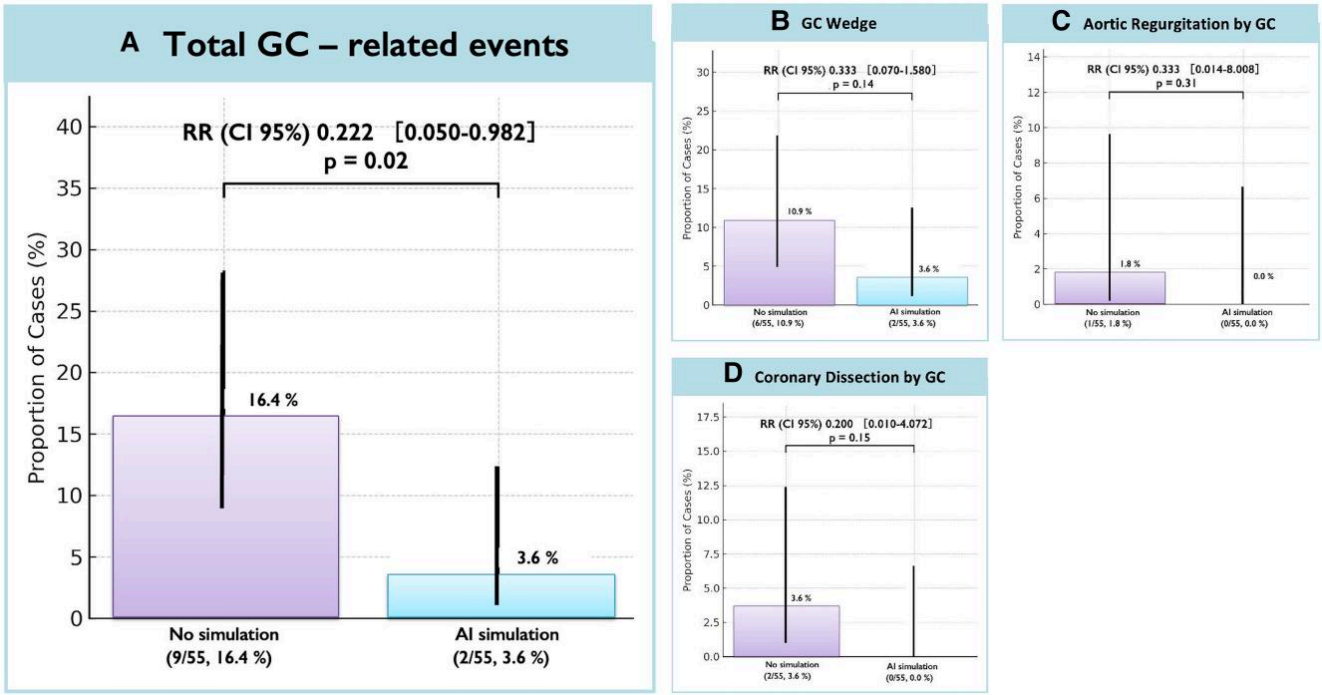
Guiding-catheter-related events. Composite catheter-related events occurred less frequently with artificial intelligence assistance, with consistent directions of effect (lower event rates) across individual component. Event definitions and adjudication are described in Methods; numerical rates and risk ratios are summarized in *Table 4*. AR, aortic regurgitation; GC, guiding catheter; ‡exploratory analysis without multiplicity adjustment.

Clinical outcomes are shown in *Table 4*. Artificial intelligence GC simulation was associated with more favourable GC-related safety indices without increasing periprocedural events. Total GC-induced events were significantly lower in the AI-simulation group (3.6 vs. 16.4%; RR 0.22, 95% CI 0.05–0.98, *P* = 0.02) (*Figure 6A*). Individual components numerically favoured AI (wedge by a GC 3.6 vs. 10.9%; AR by a GC 0 vs. 1.8%; coronary dissection by a GC 0 vs. 3.6%) (*Figure 6B–D*), though subgroup comparisons were underpowered. Other PCI-related complications, including slow-flow/no-reflow (3.6 vs. 5.4%),^17^ side-branch occlusion (1.8% in both groups),^18^ and wire perforation (0 vs. 1.8%; *P* = 0.31) were infrequent and similar between groups. There were no cases of cardiac tamponade, or emergent MCS use. The hospital length of stay and in-hospital MACCE (death, myocardial infarction, stroke, emergent target vessel revascularization, stent thrombosis) were zero in both groups. Total PCI material costs did not differ between the AI-simulation and non-simulation groups (¥635 600.5 ± 332 263.2 vs. ¥672 933.4 ± 280 104.7; *P* = 0.61).

**Table 4:** Safety outcomes and resource use

Variables	No simulation group (*N* = 55)	AI simulation group (*N* = 55)	RR (95% CI)	*P*-value
Length of hospitalization, days	3.7 ± 3.1	3.2 ± 1.7		0.29
Mechanical circulatory support, *n* (%)				
IABP				0.55
Pre-procedure support	0 (0.0)	2 (3.6)		
Bailout	1 (1.8)	0 (0.0)		
IMPELLA	0 (0.0)	0 (0.0)		N/A
VA-ECMO	0 (0.0)	0 (0.0)		N/A
Guiding catheter–induced events, *n* (%)	9 (16.4)	2 (3.6)	0.222 (0.050, 0.982)	0.02
Guiding catheter–induced coronary dissection	2 (3.6)	0 (0.0)	0.200 (0.010, 4.072)	0.15
Guiding catheter–induced aortic regurgitation	1 (1.8)	0 (0.0)	0.333 (0.014, 8.008)	0.31
Wedge position of the guiding catheter tip	6 (10.9)	2 (3.6)	0.333 (0.070, 1.580)	0.14
Complications during the procedure, *n* (%)	5 (9.1)	3 (5.4)		0.53
Slow flow or no reflow	3 (5.4)	2 (3.6)	0.667 (0.116, 3.835)	
Side branch occlusion	1 (1.8)	1 (1.8)	1.000 (0.064, 15.588)	
Vessel perforation by the guidewire	1 (1.8)	0 (0.0)		
Cardiac tamponade	0 (0.0)	0 (0.0)		
Total PCI material cost (JPY)	672 933.4 ± 280 104.7	635 600.5 ± 332 263.2		0.61
In-hospital MACCE, *n* (%)				
Cardiac death	0 (0.0)	0 (0.0)		N/A
Non-cardiac death	0 (0.0)	0 (0.0)		N/A
QMI	0 (0.0)	0 (0.0)		N/A
Non-QMI	0 (0.0)	0 (0.0)		N/A
Emergent target vessel revascularization with PCI or CABG	0 (0.0)	0 (0.0)		N/A
Thrombosis	0 (0.0)	0 (0.0)		N/A
Stroke	0 (0.0)	0 (0.0)		N/A

GC-related events, periprocedural events and cost.

CABG, coronary artery bypass grafting; IABP, intraaortic balloon pumping; MACCE, major adverse cardiac and cerebrovascular event(s); MI, myocardial infarction; PCI, percutaneous coronary intervention; QMI, Q-wave myocardial infarction; RR, relative risk; VA-ECMO, veno-arterial extracorporeal membrane oxygenation.

### Adherence and sensitivity analyses

In the AI-simulation group, the finally selected guiding catheter matched the top AI recommendation in 63.6% of cases, matched an alternative AI suggestion in 27.3%, and deviated in 9.1% (see Supplementary material online, *Table S1*). The primary outcome benefit was consistent across full- and partial-match strata without evidence of interaction (*P* = 0.42). In multivariable analyses adjusting for age, prior heart failure, SYNTAX score, lesion length, access site, operator experience, and calendar month, AI simulation was associated with significantly shorter GC engagement time (adjusted mean difference −73.6 s; 95% CI −144.0 to −3.3; *P* = 0.040) and fewer composite GC-related events (adjusted odds ratio 0.135; 95% CI 0.033–0.557; *P* = 0.0057). Effects on total procedure time, radiation exposure, and contrast volume were attenuated and no longer statistically significant, confirming robustness of the main findings (see Supplementary material online, *Table S2*). Likewise, IPTW analyses showed concordant effect estimates for procedural efficiency and safety endpoints but underpowered, with wide CIs; full results are provided in Supplementary material online, *Table S3*.

## Discussion

This study provides the first clinical evaluation of GUIDEYE, an AI-based pre-procedural simulation that assists guiding catheter selection from coronary CT. Despite comparable baseline risk, AI assistance was associated with a significantly shorter procedure time, as well as shorter GC engagement, fewer catheter exchanges, lower rates of reported device-delivery difficulty, less need for guide-extension, and lower radiation and contrast exposure, while maintaining 100% procedural success and no in-hospital MACCE. These findings support the utility and safety of AI-guided GC selection and suggest a role in improving PCI efficiency.

### Interpretation and mechanisms

Conventional GC choice rests largely on operator experience. GUIDEYE quantitatively characterizes patient-specific three-dimensional anatomy from the aorta to the coronary ostium and predicts GCs that are likely to provide greater backup and coaxiality. In this study, AI assistance was associated with fewer empirical catheter exchanges and shorter engagement and total procedure times, suggesting that anatomically tailored pre-procedural planning may streamline catheter manipulation. The pattern of shorter procedures together with lower radiation exposure and contrast use is consistent with a more efficient workflow, although residual confounding cannot be excluded. The lower rates of operator-reported device-delivery difficulty and guide-extension use are also compatible with more stable system support when AI-suggested GCs are selected. Several factors may underlie these associations: (i) less difficulty in device passage due to more favourable GC support and coaxiality, (ii) fewer GC exchanges following an initial mismatch, (iii) less time required for GC engagement, and (iv) less need for auxiliary measures such as guide-extension catheters or small balloons when adequate support is achieved from the outset. The application also estimates the likelihood that a selected guiding catheter will deeply engage or cause pressure damping based on ostial angulation and aortic root dimensions. When a high damping risk is predicted, the software simply *flags* this to the operator and displays options such as downsizing, selecting a less aggressive shape, or *optionally* choosing a side-hole catheter. The final catheter type, including any use of side holes, remained entirely at the operator’s discretion.

### Clinical implications

Efficient catheter engagement is critical in PCI, particularly in patients with complex coronary anatomy. Our findings suggest that AI simulation may be associated with immediate procedural advantages by predicting catheter shape and trajectory, thereby potentially facilitating smoother engagement. In addition to efficiency, the observed lower radiation and contrast exposure may have meaningful patient safety implications, particularly for patients with renal dysfunction or those undergoing repeated interventions. The incidence of guiding catheter-related events was significantly lower in the AI simulation group compared with the control group (3.6 vs. 16.4%, RR 0.22; 95% CI 0.05–0.98; *P* = 0.02), underscoring the potential safety advantage of pre-procedural AI planning (*Table 4*). Furthermore, the observed lower rate of guiding catheter-related events is consistent with a potential role for AI in enhancing procedural safety. Beyond direct procedural benefits, AI simulation may serve as a valuable educational tool. By providing operators with a pre-procedural plan, including catheter choice and engagement strategy, AI technology can accelerate the learning curve for junior interventionalists. This standardization may reduce variability in operator performance and promote safer PCI practice across diverse clinical settings. Side-hole use was more frequent in the AI-simulation group (80 vs. 60%; *P* = 0.02). This difference likely reflects operator preference and does not imply that side-holes are protective, particularly for the left main.

### Study limitations

#### Robustness of findings

Although this pilot trial was conducted at a single high-volume centre using a non-randomized design, the primary outcomes remained robust after adjustment for baseline imbalances, operator experience, and temporal trends. Sensitivity analyses using both multivariable regression and propensity score–based weighting consistently supported the observed between-group differences, with lower procedure time, radiation exposure, and contrast volume in the AI-simulation group, underscoring the internal validity of the findings. Finally, because the decision to use GUIDEYE was not randomized and depended on calendar period and operator use, residual selection bias and unmeasured confounding cannot be excluded.

#### Generalizability

The study setting—an experienced centre with routine IVUS guidance—likely minimized variability and strengthened internal control, but may limit generalizability to lower-volume institutions or less experienced operators. Importantly, adherence analyses demonstrated that clinical benefits were preserved even when operators deviated partially from the top AI recommendation, suggesting potential applicability in real-world environments. Future multicentre studies involving a broader range of operators and practice settings are warranted to confirm external validity and quantify training, educational benefits, and assess cost-effectiveness.

#### Statistical power

With 55 vs. 55 patients, the study was adequately powered to detect the observed difference in the primary endpoint but underpowered for rare safety events, as reflected by wide CIs for individual GC-related events. A formal prospective power calculation and an adequately powered multicentre study are warranted to confirm safety effects and health-economic endpoints. *Post hoc* calculations (see Supplementary material online, *Table S4*) indicate that ∼77 patients per group would be required to detect a difference in GC-related event rates between 16.4 and 3.6% with 80% power at a two-sided *α* of 0.05. Strengths include prospective enrolment, uniform angiography platform, universal IVUS guidance, and objective, quantitative endpoints. Limitations include single-centre analysis of a two-centre registry with a historical control, leaving room for residual confounding; modest sample size; and lack of per-case adherence measurement to AI recommendations. Rare safety events were underpowered, as reflected by wide CIs. In one AI-group case, severe descending aortic tortuosity during femoral PCI necessitated GC exchange, highlighting algorithmic limits in some anatomies. Selection and temporal biases remain possible despite uniform IVUS and a single imaging platform; rare safety events were underpowered. These findings, while robust across sensitivity analyses, should be interpreted with caution because adjusted effects on several efficiency endpoints were attenuated and the study was underpowered for rare safety events. Although CT-based AI assistance was associated with shorter total procedure time, the magnitude of this difference likely reflects not only shorter engagement but also unmeasured differences in case mix, temporal trends, and operator behaviour. Despite adjustment for key clinical and procedural covariates, residual confounding cannot be excluded, and our findings should be interpreted as associative rather than causal.

### Future directions

A multicentre, randomized trial—powered for clinical and health-economic endpoints and incorporating adherence tracking—is warranted. Technically, continued model training may improve accuracy and broaden scope from GC choice to wire/balloon/stent selection and strategy for complex lesions (e.g. bifurcations, CTO). The integration of pre-procedural three-dimensional CT with intraprocedural angiography could enable real-time navigation. Such advances may ultimately extend to structural interventions, including left atrial appendage closure (LAAC) and transcatheter aortic valve implantation.^19,20^ In addition, integration of such CT-based AI simulations into live case transmissions at conferences could standardize how guiding-catheter selection is discussed and demonstrated, thereby enhancing educational impact for trainees and practising operators.

## Conclusion

Pre-procedural, CT-based, automated AI guiding-catheter simulation was associated with shorter procedure time and a favourable procedural efficiency and safety profile in this single-centre observational registry. By standardizing GC selection and being associated with fewer empirical catheter exchanges, AI simulation may function as a ‘procedural equaliser’ that helps mitigate differences in operator experience; this hypothesis warrants confirmation in randomized multicentre trials.

## Lead author biography


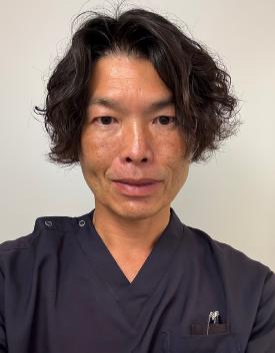


Masataka Yoshinaga was born in 1978 in Fukuoka, Japan. He graduated from Fujita Health University in 2006 and is currently serving as a Lecturer at Fujita Health University Bantane Hospital in Aichi, Japan. His main areas of expertise and interest are percutaneous coronary intervention (PCI) and endovascular treatment (EVT).

## Supplementary Material

ztag021_Supplementary_Data

## Data Availability

The data that support the findings of this study are available from the corresponding author upon reasonable request. De-identified individual participant data, the data dictionary, and analysis code will be shared for academic purposes after approval of a methodologically sound proposal.
