# Supplementary material for: Pilot clinical evaluation of artificial intelligence–driven guiding catheter simulation for optimizing percutaneous coronary intervention
Source: Eur Heart J Digit Health. 2026 Feb 3;7(2):ztag021. doi: 10.1093/ehjdh/ztag021 (PMC12897535; doi:10.1093/ehjdh/ztag021)
Supplement: ztag021_Supplementary_Data [file ztag021_supplementary_data.docx]

# Supplementary Material

## Statistical Analysis Plan

To mitigate selection and temporal biases inherent to a single-center, nonrandomized design with historical controls, we prespecified exploratory sensitivity analyses adjusting for baseline imbalances and time trends. Multivariable models for continuous outcomes (procedure time, radiation, contrast) adjusted for age, prior heart failure, SYNTAX score, lesion length, access site, and operator experience (categorical), with calendar month included as a covariate. Categorical outcomes (GC exchanges, device delivery difficulty, GC-related events) were tested with logistic regression using robust standard errors. As an additional bias-reduction strategy, propensity score–based inverse probability of treatment weighting (IPTW) was applied.

## Table S1. Adherence to AI Recommendations

For each case in the AI-simulation group, adherence to AI recommendations was classified as: full match (final GC matched the top AI recommendation), partial match (final GC matched an alternative suggestion), or no match (final GC deviated). In this study, adherence was as follows: Top recommendation: 35/55 (63.6%), Alternative suggestion: 15/55 (27.3%), Deviation: 5/55 (9.1%).

| Concordance Category | Definition | n (%) |
| --- | --- | --- |
| Top recommendation | Final guiding catheter fully matched the top AI recommendation (shape/size) | 35 (63.6%) |
| Alternative suggestion | Final guiding catheter matched one of the alternative AI suggestions | 15 (27.3%) |
| Deviation | Final guiding catheter deviated from all AI recommendations | 5 (9.1%) |
| Total |  | 55 (100%) |

## Table S2. Adjusted Outcomes (Multivariable Models)

Results of multivariable adjusted analyses for primary and secondary outcomes.Adjusted models controlled for age, prior heart failure, SYNTAX score, lesion length, access site, operator experience, and calendar month.

| Outcome | Adjusted Mean/OR | 95% CI (Lower) | 95% CI (Upper) | p-value |
| --- | --- | --- | --- | --- |
| GC engagement time (s) | -73.6 | -144.0 | -3.3 | 0.040 |
| Procedure time (min) | -5.8 | -17.9 | 6.3 | 0.34 |
| Radiation dose (mGy) | -85.2 | -243.8 | 73.4 | 0.28 |
| Contrast volume (mL) | -12.6 | -36.1 | 10.9 | 0.29 |
| GC-related events (OR) | 0.135 | 0.033 | 0.557 | 0.0057 |

## Table S3. Sensitivity Analyses (IPTW)

Results of inverse probability of treatment weighting (IPTW) analyses for primary and secondary outcomes. Effect estimates were directionally consistent but with wide confidence intervals, reflecting limited power.

| Outcome | Adjusted Mean/RR | 95% CI (Lower) | 95% CI (Upper) | p-value |
| --- | --- | --- | --- | --- |
| GC engagement time (s) | -68.2 | -152.5 | 16.1 | 0.11 |
| Procedure time (min) | -4.9 | -18.2 | 8.4 | 0.47 |
| Radiation dose (mGy) | -77.5 | -268.1 | 113.2 | 0.42 |
| Contrast volume (mL) | -10.8 | -40.2 | 18.5 | 0.46 |
| GC-related events (OR) | 0.198 | 0.041 | 0.954 | 0.043 |

## Table S4. Post‑hoc Power Calculation for GC‑related events

Based on a two-sided α of 0.05 and 80% power, a sample size of approximately 77 patients per group (154 in total) is required to detect a reduction in GC-related events from 16.4% to 3.6%.

| Parameter | Value |
| --- | --- |
| Control event rate | 16.4% |
| AI event rate | 3.6% |
| α / Power | 0.05 (two‑sided) / 0.80 |
| Required n per group | ≈77 |

Notes:

• Effect estimates are reported as risk ratios (RR) with 95% confidence intervals for categorical outcomes, and adjusted mean differences with 95% CI for continuous outcomes.

• Inverse probability of treatment weighting (IPTW) was generated using stabilized weights, and covariate balance was assessed by standardized mean differences (<0.1 considered adequate).

• Table S4 power estimates were calculated post hoc using a two-sided α=0.05 and 80% power, based on observed event rates (control: 16.4%, AI: 3.6%).
